# Comparative Analysis of Amino Acid and Biogenic Amine Compositions of Fermented Grape Beverages

**DOI:** 10.3390/metabo13080892

**Published:** 2023-07-27

**Authors:** Erdenetsetseg Nokhoijav, Andrea Guba, Uladzislau Vadadokhau, József Tőzsér, Zoltán Győri, Gergő Kalló, Éva Csősz

**Affiliations:** 1 Proteomics Core Facility, Department of Biochemistry and Molecular Biology, Faculty of Medicine, University of Debrecen, 4032 Debrecen, Hungary; erdenetsetseg.n@med.unideb.hu (E.N.); guba.andrea@med.unideb.hu (A.G.); uladzislau.vadadokhau@med.unideb.hu (U.V.); tozser@med.unideb.hu (J.T.); kallo.gergo@med.unideb.hu (G.K.); 2 Doctoral School of Molecular Cellular and Immune Biology, University of Debrecen, 4032 Debrecen, Hungary; 3 Institute of Nutrition, Faculty of Agricultural and Food Sciences and Environmental Management, University of Debrecen, 4032 Debrecen, Hungary; gyori.zoltan@unideb.hu

**Keywords:** amino acid, biogenic amine, wine, wine vinegar, LC-MS

## Abstract

Amino acids and biogenic amines are important components of food and beverages. In grape-derived products such as wine and wine vinegar, they can have different origins and can influence the odor and taste of the products. Their concentration is influenced by the grape variety, vintage, and winemaking process. In our study, we carried out an LC-MS-based comparative analysis of 22 grape-derived beverages, including three different wine types and four wine vinegar samples from the Tokaj region in Hungary. The concentrations of 23 amino acids and 10 biogenic amines were examined, and the differences among the sample types were analyzed. The differences in the concentrations of some metabolites between Aszú–Furmint pairs originating from the same wineries and year provide information on the effect of botrytized grape on wine composition. Our data can provide further evidence on how the production process shapes the metabolite content of beverages and highlight the nutritional value of wine vinegar.

## 1. Introduction

Grape is the principal raw material in making wines; in addition to that, it can be consumed as grape juice or vinegar.

The Tokaj wine region, announced as a UNESCO world heritage area in 2002, is famous for its high-quality, unique style of wines. One of its most used grape varieties for winemaking is Furmint, used for Furmint wine, which ranges from dry to sweet wines [1]. Aszú wine is produced from Furmint wine poured over grape berries infected with *Botrytis cinerea* in a process called noble rotting. Due to the presence of botrytized grapes, Aszú is shaped with its unique taste and specific characteristics, such as consistency and sweetness resulting from the high residual sugars of grapes formed during the noble rotting [2,3,4]. Winemakers also produce Essence, an elegant form of wine with high sugar content and a honey-like consistency [4]. For Essence, the noble-rotten berries are not pressed, and the juice generated by the inner pressure of the berries is fermented slowly over time [2,3,5].

Besides wines, another grape-derived product is vinegar. Vinegar is an acetic acid solution obtained from various sources after the fermentation of grape must by *Acetobacter* sp. bacteria [6].

In enology, the study of wine, analysis of amino acids and biogenic amines has been widely conducted concerning health benefits, nutritional values, quality, and hygienic issues [7,8,9,10,11,12,13].

Amino acids present in wine and other grape-derived fermented products can play a crucial role in the growth of yeast and lactic acid bacteria as a nitrogen source during fermentation [14]. In total, 30–40% of total nitrogen in wine is found in amino acids [15,16], and the amino acid profile can be a quality fingerprint of wine and can contribute to its odor and flavor [17,18]. Proline, glutamate, and arginine are the most abundant amino acids in wine [19,20,21], and according to some studies, the high concentrations of proline and other amino acids such as alanine, phenylalanine, aspartate, and threonine can be involved in wine aroma formation and appearance of the unique taste as well as the color of wine [18]. The concentrations of the amino acids in wine can be determined by different factors: the grape-variety-specific amino acid content of grape juice, the speed of the utilization of amino acids by yeast, and their metabolism during fermentation [22].

Biogenic amines are produced during the fermentation of beverages as a result of the decarboxylation of their precursor amino acids [13] or can be present in the grape or raw material itself [1,19,23,24,25].

The composition and the presence of amino acids, biogenic amines, and other nutritious components in grape-derived products are mostly related to the grape variety, weather conditions, and production process [16,26,27]. Before or during the fermentation, these compounds may come in contact with different microbial agents such as *Botrytis cinerea*, required for the noble rotting of grapes [2,3,4]; *Saccharomyces cerevisiae*, responsible for alcoholic fermentation [28]; and *Acetobacter* sp., used for vinegar production [6].

Even though they might have a similar origin, the amino acid and biogenic amine compositions of wine (Furmint), botrytized wine (Aszú), Essence, and wine vinegar can substantially differ from each other.

Liquid chromatography (LC) and mass spectrometry (MS) methods are widely used for the qualitative and quantitative analysis of wine and grape-derived products [29,30,31,32,33,34]. To the best of our knowledge, no comparative studies on amino acids and biogenic amines in these grape-derived product types have been conducted so far. In our study, by applying ultra-performance liquid chromatography (UPLC) and mass spectrometry (MS), we aimed to fill this gap and provide a comprehensive LC-MS-based comparative analysis of amino acids and biogenic amines in wine, Essence, and wine vinegar.

## 2. Materials and Methods

### 2.1. Reagents

All reagents were purchased from Sigma Aldrich (St. Louis, MI, USA) if not stated otherwise. A standard mixture of 17 amino acids, namely histidine, serine, arginine, glycine, aspartate, glutamate, alanine, threonine, proline, cysteine, lysine, tyrosine, methionine, valine, isoleucine, leucine, and phenylalanine, was acquired from Waters (Milford, MA, USA). Asparagine, glutamine, taurine, histamine, ethanolamine, methylamine, citrulline, ethylamine, ornithine, putrescine, serotonin, cadaverine, tyramine, tryptamine, and 2-phenylethyl-amine standards were obtained from Sigma-Aldrich (St. Louis, MI, USA), and tryptophan was purchased from Cambridge Isotope Laboratories (Tewksbury, MA, USA).

### 2.2. Samples

Four types of wine-derived beverages, including Aszú wine (*n* = 8), Furmint wine (*n* = 8), Essence (*n* = 2), and wine vinegar (*n* = 4) from the Hungarian Tokaj region, a UNESCO World Heritage site, were selected for examination. The samples were from different wineries and years (see Table S2).

### 2.3. Sample Preparation

In total, 200 µL of the sample from each beverage was transferred to a Nanosep 3 kDa (Pall Corp, New York, NY, USA) size-exclusion spin column to separate the small molecules from the proteins and molecules with higher molecular weight. Samples were centrifuged with 12,800× *g* at 4 °C, 3 times for 10 min, and the flow-through was completely dried in a vacuum concentrator (ThermoScientific, San Jose, CA, USA).

### 2.4. Derivatization

The dried samples were re-dissolved in 70 µL borate buffer (pH 8.8), and the pH was adjusted to pH ≥ 8 with 2 M NaOH, followed by the final sample volume adjustment to 80 µL with borate buffer. The sample was subjected to derivatization by adding 20 µL AccQ-Tag Ultra reagent (Waters, Milford, MA, USA) and incubated at 55 °C for 10 min [35]. The derivatizing agent used was 6-aminoquinolyl-N-hydroxysuccinimidyl carbamate, which reacts with both primary and secondary amines in a very quick reaction, forming stable derivatives. Excess AQC reagent reacts with water to form 6-aminoquinoline (AMQ). Calibration standards containing each analyte were prepared and used to record an 10-point calibration curve in the concentration range of 0.5–30 pmol/µL for each analyte [36]. The calibration standards and the derivatized samples were subjected to LC-MS analyses.

### 2.5. UPLC-UV and MS Analysis

Two technical replicates were analyzed by injecting 1 µL of sample into Acquity H-class UPLC (Waters, Milford, MA, USA) system. Liquid chromatographic separation was performed using an 11 min gradient on an Acquity H-class UPLC system equipped with a PDA detector (Waters, Milford, MA, USA). The separation of the derivatized amino acids was carried out on an AccQ-tag Ultra C18 column (1.7 µm; 2.1 × 100 mm, Waters, Milford, MA, USA) guarded by an Acquity in-line filter (0.2 µm; 2.1 mm, Waters, Milford, MA, USA). The flow rate was set to 0.65 mL/min, and the column temperature was set to 54 °C. Solvent A was 100% AccQ-tag Ultra eluent A, solvent B was 10% AccQ-tag Ultra eluent B in LC water, solvent C was LC water, and solvent D was 100% AccQ-tag Ultra eluent B. UV spectra were recorded at 260 nm with 10 points/s sampling rate, and the chromatograms (Figure S1)were evaluated with Empower software (version 3.0, Waters, USA). In the case of wine vinegars, besides the UV chromatograms, MRM/SRM scans were recorded on a 5500 QTRAP mass spectrometer (Sciex, Framingham, MA, USA) controlled by Analyst software (version 1.6.3., ABSciex, Framingham, MA, USA). Electrospray ionization with 5500 V spray voltage was applied, and SRM spectra were recorded in positive ion mode. During the analysis, the ion source gas 1 was set to 30 psi; the ion source gas 2 was set to 50 psi; the curtain gas was set to 30 psi; and the source temperature was 500 °C. The spectra were examined using Skyline (v.20.2, www.maccosslab.org, accessed on 21 January 2022). The applied gradient and the MRM/SRM transitions used were described earlier [36]. The concentration of each analyte was given as the mean of the two technical replicates.

### 2.6. Statistical Analysis

Descriptive statistics followed by Mann–Whitney test was performed using Graph Pad Prism software (version 8.0.1 for Windows, GraphPad Software, San Diego, California USA, www.graphpad.com, accessed on 21 January 2022). The *p*-value of *p* ≤ 0.05 was considered statistically significant. Principal component analysis (PCA) was computed by the prcomp function of the R stats package. The first three principal components were extracted, and 3D plot was generated utilizing the plotly library in R.

## 3. Results and Discussion

The examination of wine and wine vinegar content can provide important information on their quality and can also indicate their potential utilization as functional food. Several studies have already examined the amino acid and biogenic amine content of white wine and wine vinegar (Table S1), but the results are not consistent all the time. The type of analytical method and instrumentation used for the examination highly influences the results. According to data published in the scientific literature, the type of grape utilized, the year of collection, and the type of microbes used for fermentation have a great influence on amino acid and biogenic amine content of grape-derived products [1,22,37]. Wine can be a rich source of amino acids and other compounds beneficial for our health [38], but due to its alcohol content, its consumption can be limited. Wine vinegar can be an alternative source of amino acids and biogenic amines, but to the best of our knowledge, no comparative analysis of wine and wine vinegar regarding their amino acid and biogenic amine content has been carried out so far.

In our study, we selected three wine types from the Tokaj region, a Hungarian wine region that is part of UNESCO World Heritage: Furmint, Aszú and Essence. We also seleected four different wine vinegars originating from grapes from the same region.

By utilizing a sensitive UHPLC-MS system, we examined the concentrations of 23 amino acids and 10 biogenic amines in eight Furmint, eight Aszú, two Essence, and four wine vinegar samples. Because both Furmint and botrytized berries are involved in the process of Aszú production, we performed a comparison of Furmint–Aszú pairs originating from the same winery and same year.

### 3.1. Examination of Amino Acids in Grape-Derived Products

**Figure 1:**
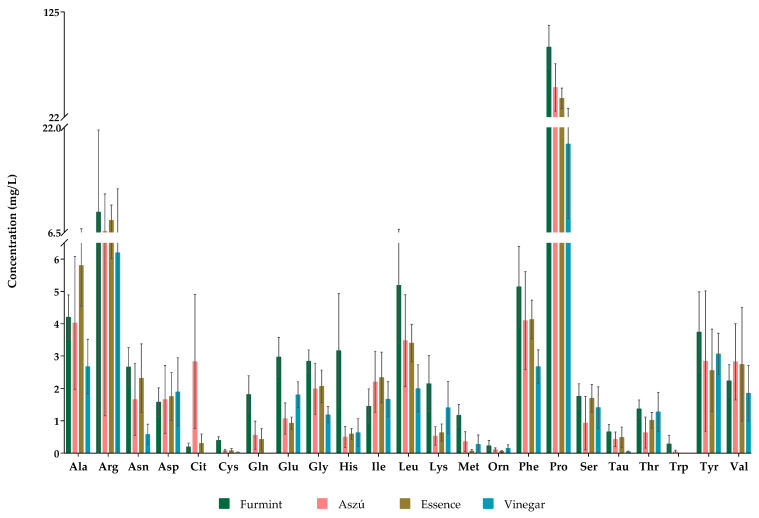
The concentrations of amino acids in all studied grape-derived beverages. The y-axis shows the mean concentrations in mg/L and standard deviations (SD) of detected amino acids.

All the examined 23 amino acids were detected and quantified in Aszú and Furmint samples, while we could not detect glutamine, citrulline, and tryptophan in wine vinegar samples (Figure 1). In Essence, all amino acids except tryptophan could be detected. Ornithine, cysteine, and methionine were detected but not quantified, as their concentrations were lower than the LOQ (Table S2).

Regarding the concentrations of the individual amino acids, proline was the most abundant amino acid in all of the examined products. The proline concentration in Aszú ranged from 19.6 to 103.7 mg/L, while in Furmint, it varied between 53.9 and 119.9 mg/L. The proline concentration in Essence was 31.5–48.9 mg/L, whereas, in wine vinegar, it varied in the range of 10–37.1 mg/L (Figure 1, Table S2).

Besides proline, arginine, alanine, and phenylalanine were present in relatively high concentrations in all studied wine samples, while arginine was one of the most abundant amino acids in vinegar. Typically, in Furmint, the levels of amino acids were higher than in the other sample types.

Our data are in accordance with data published in the scientific literature. Csomos et al. found proline and arginine as the amino acids with highest concentration in Tokaji Aszú and Szamorodni samples [39]. Similar trends were observed by other groups; however, the exact concentration values are different. Kutlan et al. [40] found high concentrations of arginine, glutamate, alanine, lysine, and aspartate in a Hungarian white wine, Badacsonyi Szürkebarát, originating from a different geographical region of Hungary. Their study focused on the optimization of the analytical method and did not examine the levels of histidine, taurine, citrulline, proline, ornithine, and cysteine. A study on amino acids in Spanish white wines by Gomes-Alonso et al. [21] found approximately 10 times higher proline concentration (904 mg/L) than we did. A similar result has been reported by Tuberoso et al. [20]: in their study the average proline concentration was 844.7 mg/L in the sherry-like Italian wine Vernaccia di Oristano. They also found that beside proline, glutamate, aspartate, and glutamine were abundant [20]. According to a study on amino acids by Bouzas-Cid et al., concentrations of proline varied in the range of 11.95–2139.78 mg/L in Portugal’s Albarino white wine [41]. Studies on amino acids in Greek white wine showed arginine as the amino acid with the highest concentration (104.3 mg/L and 199 mg/L) [19,37]. Proline, glycine, alanine, arginine, and threonine were found to be the most abundant amino acids, representing about 75% of the total amino acids in the balsamic vinegar of Modena, Italy [12].

Scientific evidence shows that the amino acid concentration is dependent on the fermentation: the type of fermenting microbes and the duration and conditions of fermentation greatly influence the amino acid content [28]. Besides the fermentation, the grape variety used has an important role in determining the amino acid content. In addition to our results, the studies listed in Table S1 indicate the importance of grape type to the amino acid content.

### 3.2. Comparative Analysis of Wine and Wine Vinegar Samples Regarding Their Amino Acid Content

**Figure 2:**
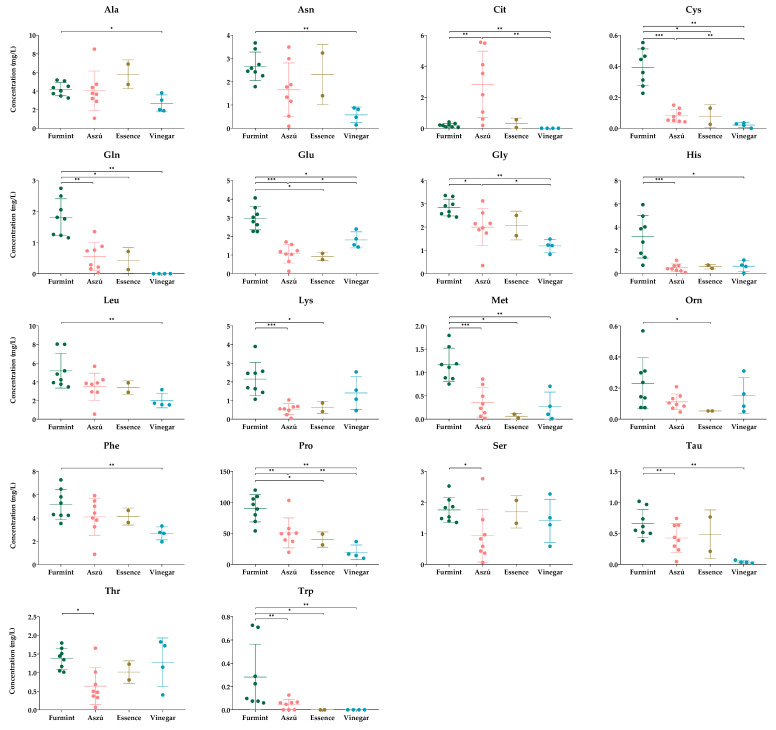
The concentrations of amino acids, showing statistically significant differences between the groups. The y-axis shows the concentrations in mg/L of individual amino acids and SD in the four types of beverages examined. Each dot represents mean value of amino acids in the samples. * *p* ≤ 0.05; ** *p* ≤ 0.01; *** *p* ≤ 0.001.

The comparative analysis between different samples showed significant differences in the concentrations of several amino acids (Figure 2).

According to the statistical analysis, 18 amino acids showed a significant difference.

When we compared the Furmint with Aszú, the levels of cysteine, glutamine, glutamate, glycine, histidine, lysine, methionine, proline, serine, taurine, threonine, and tryptophan were significantly higher and the citrulline level was significantly lower in Furmint. Regarding the comparison between Furmint and Essence, the concentrations of cysteine, glutamine, glutamate, lysine, methionine, ornithine, tryptophan, and proline were significantly higher in Furmint. Both Aszú and Essence are made from botrytized grapes, and as we expected, we could observed similar amino acid profiles in the two sample types.

Wine vinegar is the product of a different type of fermentation, and this difference was only partially reflected in the levels of amino acids. We expected a marked difference between the wine and wine vinegar samples; however, based on our data, the wine vinegar amino acid content in the case of most of the amino acids was very similar to the amino acid content of wines (Figure 2). This result highlights the utility of wine vinegar as a functional food with no alcohol content.

Comparing the vinegar samples to Furmint, the levels of alanine, asparagine, citrulline, cysteine, glutamine, glutamate, glycine, histidine, leucine, methionine, phenylalanine, proline, tryptophan, and taurine were higher in Furmint. Based on the comparative analysis between wine vinegar and Aszú samples, cysteine, glycine, and proline concentrations were significantly lower, whereas the glutamate level was higher in wine vinegar. At the same time, no statistically significant difference between Essence and Vinegar samples could be detected.

Because the type of grape and fermentation conditions differ among the three examined wine types, our results further highlight, besides the importance of grape type, the differential utilization of amino acids by *Acetobacter* species during the acetic fermentation and *Saccharomyces* species during the alcoholic fermentation.

Overall, these findings provide valuable insights into the composition of amino acids in different wine and wine vinegar samples and might indicate the importance of understanding their potential effects on the taste and aroma of these products.

### 3.3. Examination of Biogenic Amines in Grape-Derived Products

Biogenic amines generated from the decarboxylation of amino acids can have very profound health effects. Some of them (putrescine, cadaverine) can have toxic effects in higher concentrations, indicating problems for food processing or storage, while others, such as histamine, can be of concern for consumers having problems with histamine degradation [42,43,44].

**Figure 3:**
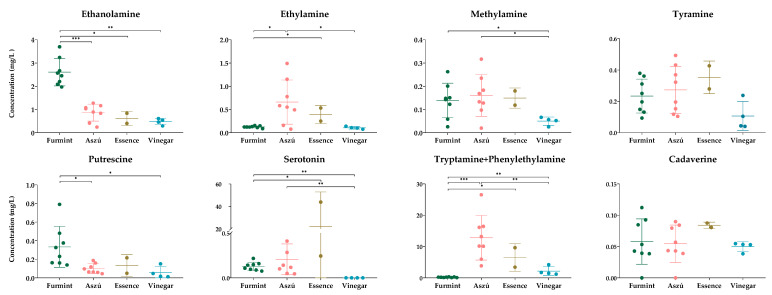
The concentrations of the biogenic amines. The y-axis shows the concentrations in mg/L of individual biogenic amines and SD in four types of beverages. Each dot represents mean value of biogenic amines in the samples. * *p* ≤ 0.05; ** *p* ≤ 0.01; *** *p* ≤ 0.001.

In order to obtain information on beverage quality, we examined the amounts of some selected biogenic amines (Figure 3).

We could detect all examined biogenic amines except histamine. This result does not exclude the presence of histamine in the samples; it only indicates that we can obtain no information about this analyte. Considering data from the scientific literature showing the presence of histamine in wine samples originating from the same or different regions, we cannot exclude that pre-column derivatization followed by chromatography is not a suitable method for histamine examination in wine and wine vinegar samples. At the moment, we do not have enough information to judge if histamine levels were under our detection limit or histamine was missing from the sample. The peaks for the tryptamine and phenylethylamine could not be separated from each other, and as such, we could obtain information only about the combined amount of these two biogenic amines. The concentrations of all examined biogenic amines were relatively low, except for one Essence sample, but in all cases, their amount was below the toxicity limits reported by the European Food Safety Authority (EFSA) for fermented food [45]. For wine and grape-derived beverages, no such guidelines are available so far, only country-specific recommendations.

Among the biogenic amines analyzed, ethanolamine, ethylamine, and tyramine were found to have the highest concentration in the samples, and these findings were consistent with previous studies [30,39,46,47].

Csomos et al. determined that tyramine, putrescine, and histamine were dominant biogenic amines in Aszú wine, while tyramine and putrescine were amongst the major biogenic amines in Szamorodni wines [39].

According to studies on Hungarian white wine, Badacsonyi Szürkebarát, and Italian white wine, ethanolamine was the biogenic amine with the highest concentration [40,46]. Another study on Italian white wines, including Chardonnay, Passerina, Pecorino, and Trebbiano, found ethylamine was the biogenic amine with the highest concentration, followed by putrescine [47]. The results, in the case of ethanolamine and ethylamine, agree with the results of Manetta et al., who identified ethanolamine, putrescine, and ethylamine as the most abundant biogenic amines in white and red wines analyzed [30]. In Sauvignon Blanc, a Chilean reserve varietal wine, putrescine showed the highest concentration and was followed by histamine and tyramine [48]. Similar results were found in case of Italian sherry-like wine, Vernaccia di Oristano [20], Italian white wines [43], Spanish white wine [49], and Greek white wines [32]. In our study, putrescine showed significantly higher concentration in Furmint than in the other three products, but this was not an abundant biogenic amine in the examined samples. According to the study performed by Torre et al., the concentrations of histamine, cadaverine, and tyramine in Sicilian white wines were near the detection limit, whereas tryptamine and phenylethylamine were not detected [50]. Their results were similar to data obtained from white wine from the Abruzzo region, Italy, and agreed with the study results by Bover-Cid et al. [30,51], indicating variations in the amount and detection depending on the type of wine analyzed and the experimental setup applied (Table S1). In our samples, serotonin was present in low concentration in all but one Essence sample, where it showed high concentration. In the majority of our reviewed studies, serotonin was not measured in wine-derived products, and those who measured it found the concentration of serotonin low in Spanish [51] and Italian white wines [43].

Regarding the wine vinegar, we could detect all examined biogenic amines but histamine and serotonin. A study of biogenic amines in white wine vinegar from Spain found putrescine and cadaverine were the biogenic amines with the highest concentration, along with spermine, while methylamine, tyramine, and phenylethylamine were not detected [52]. We could quantify all of these biogenic amines and found that the concentrations of cadaverine, methylamine, putrescine, and tyramine are the lowest in wine vinegar among all our examined samples. Kutlan et al. found ethylamine in the highest concentration in a Hungarian wine vinegar [40]. According to our results, in the examined wine vinegar samples, tyramine had the highest concentration, followed by ethanolamine.

### 3.4. Comparative Analysis of Wine and Wine Vinegar Samples Regarding Their Biogenic Amine Content

In order to examine the differences among the different grape-derived beverages, statistical analysis was performed (Figure 3). We could detect statistically significant changes in the levels of five biogenic amines between the different sample types. Because the individual amounts of tryptamine and phenylethylamine could not be determined, we omitted these two biogenic amines from further examinations in spite of their combined concentration showing statistically significant differences between the studied groups.

Comparing the Furmint samples to Aszú, significantly higher concentrations of ethanolamine and putrescine and lower concentration of ethylamine was found in Furmint. Between Furmint and Essence, we could find a statistically significant difference in the cases of serotonin, ethanolamine, and ethylamine: the concentration of ethanolamine was higher in Furmint, while the concentrations of serotonin and ethylamine were lower. Wine vinegar had the lowest concentrations of cadaverine, methylamine, putrescine, and tyramine among all the samples. The levels of ethanolamine, methylamine, putrescine, and serotonin were higher in Furmint, while the levels of ethylamine, methylamine and serotonin were higher in Aszú when compared to wines.

### 3.5. Discrimination of Sample Types Based on Their Amino Acid and Biogenic Amine Content

**Figure 4:**
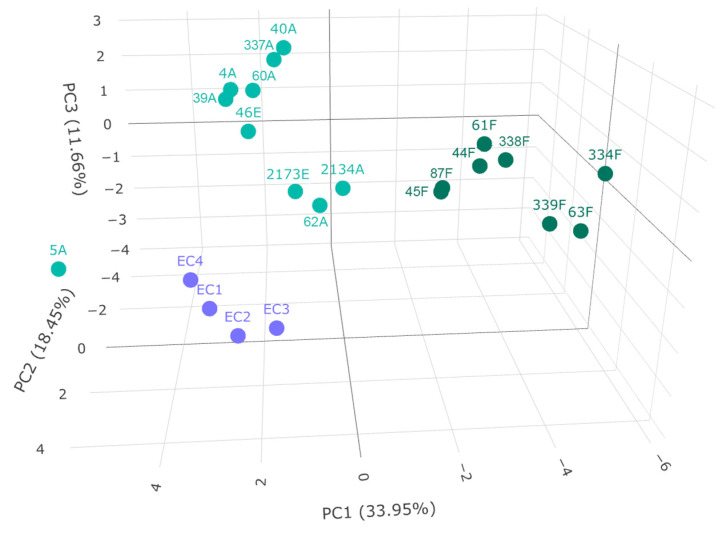
Principal component analysis of grape-derived beverages. Variance explained by principal components is indicated in axes labels. The samples in dark green refer to Furmint, the ones in light green to Aszú and Essence, and the ones in purple to wine vinegar.

In order to examine if the sample groups can be discriminated based on their amino acid and biogenic amine content, principal component analysis was carried out (Figure 4).

Three groups could be discriminated; Furmint samples formed a distinct group, as well as wine vinegar samples. The Aszú and Essence samples formed another group, but the Aszú and Essence samples could not be separated from each other, which is expected in the light of their manufacturing process. These results are in line with the ones obtained by statistical analysis (Figure 2 and Figure 3). Sample 5A is an outlier, which is surprising, as the values for the individual analytes are not outliers per se. This was a 5-basket Aszú from 2000.

Two subgroups could be identified in the case of both Aszú and Furmint samples. In the Aszú group, one subgroup contained two Aszú and one Essence sample, where the Aszú 2134A and Essence 2173E were from 1940. In the case of Furmint samples, one subgroup contained wines from 2013 except 87F, which was from 2016, while the other subgroup contained two wines from 2017 and one from 2008. The wineries could not be discriminated, and the terroir information was not available in all cases.

Sample types could be discriminated, but very likely more samples should be analyzed and included to be able to make a better distinction between the samples based on year, winery, and terroir.

### 3.6. Examination of the Effect of Botrytized Grapes on Wine Metabolite Content

**Figure 5:**
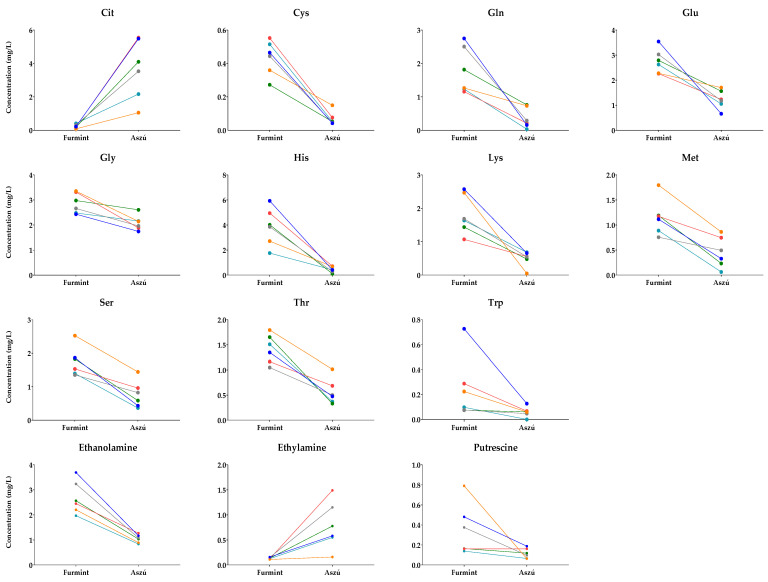
Comparative analysis of Furmint–Aszú pairs. The y-axis shows the concentrations in Furmint and Aszú in mg/L of those amino acids and biogenic amines for which statistically significant difference could be observed. Colored dots, connected with lines, represent mean values of the analytes in the paired samples.

Pairs of Aszú and Furmint originating from the same year and winery were selected (Table S2), and their metabolite content was examined. In these cases, the only difference between the Aszú and Furmint samples was the presence of botrytized grapes in the Aszú samples. The paired *t*-test results show statistically significant differences in the concentrations of 11 amino acids and 3 biogenic amines between Furmint and Aszú sample pairs (Figure 5).

The concentrations of analytes shown in Figure 5 changed in the same direction, indicating the influence of botrytized grapes on these metabolites. The higher concentrations in Furmint were dominant; however, for citrulline and ethylamine, we could observe an increasing trend in Aszú compared to Furmint.

For the other examined metabolites, the directions of changes were mixed, suggesting that other factors might be responsible for the observed concentration changes.

Our data suggest that the presence of botrytized grapes in Aszú samples has a significant impact on the amino acids and biogenic amine content compared to Furmint samples from the same winery and year. These results demonstrate the unique nature of Aszú and Furmint wines and the significant role of botrytized grapes in shaping their metabolite concentration.

## 4. Conclusions

A comparative analysis of the concentrations of amino acids and biogenic amines in different types of grape-derived beverages was carried out. The differences in the levels of amino acids and biogenic amines measured in the Essence, Aszú, and Furmint wine and wine vinegar originating from the same geographical region can reflect the differences in the production process. By comparing the Aszú–Furmint pairs originating from the same winery and year, we could identify amino acids that are sensitive to the difference in the production process, namely the presence of botrytized grapes in Aszú. Comparing the amino acid and biogenic amine content of wine vinegar and wines, all amino acids but citrulline, glutamine, and tryptophan were in the same range. The metabolite content of the examined samples highlights the importance of beverage processing and the presence of botrytized grape in determining the amino acid and biogenic amine content of the studied grape-derived beverages.

## Data Availability

We encourage all authors of articles published in MDPI journals to share their research data. Spectral chromatograms are available by asking authors. Data is not publicly available due to privacy.

## Supplementary Materials

The following supporting information can be downloaded at https://www.mdpi.com/article/10.3390/metabo13080892/s1. Figure S1: Representative chromatograms of the 33 derivatized amino acids and biogenic amines in the examined products; Table S1: The concentrations (mg/L) of amino acids and biogenic amines in wine and wine vinegar samples found in scientific literature as reported by various groups; Table S2: The concentrations (mg/L) of amino acids and biogenic amines.
