# Supplementary material for: Comparative Analysis of Amino Acid and Biogenic Amine Compositions of Fermented Grape Beverages
Source: Metabolites. 2023 Jul 27;13(8):892. doi: 10.3390/metabo13080892 (PMC10456964; doi:10.3390/metabo13080892)
Supplement: Supplementary file 1 [file metabolites-13-00892-s001.zip › metabolites-2513780-supplementary/Figure S1.pdf]

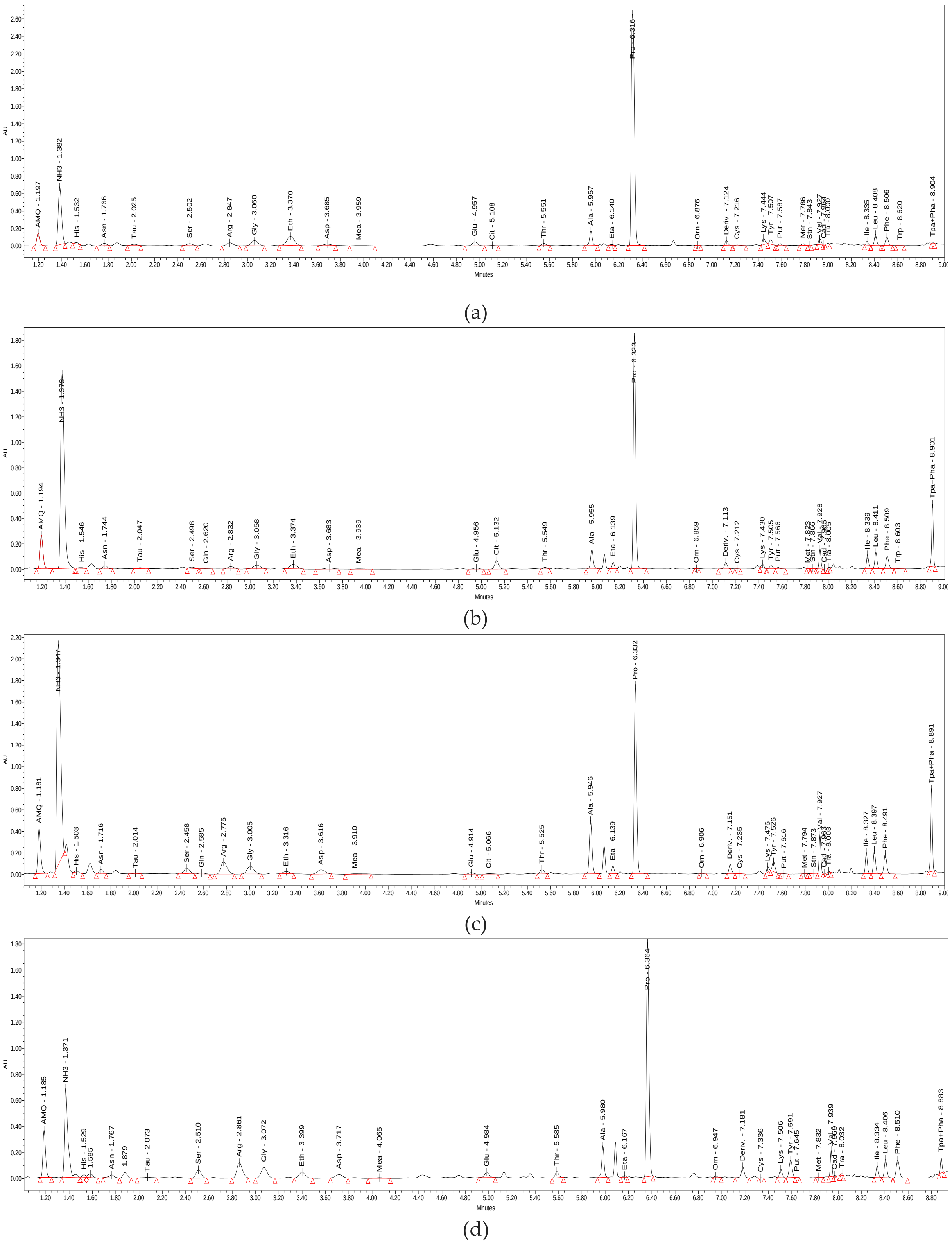

(a) (b) (c) (d)

Figure S1. Representative chromatograms of the 33 derivatized amino acids and biogenic amines in the examined products. (**a**) Furmint; (**b**) Aszú; (**c**) Essence; (**d**) Vinegar. The *x* axis shows the retention time in minutes while the *y* axis shows the intensity in form of arbitrary units (AU) registered in the PDA detector at 260 nm. For amino acids the three-letter code was used, AMQ: 6-aminoquinoline, Tau: taurine, Hsn: histamine, Eth: ethanolamine, Mea: methylamine, Eta: ethylamine, Derivatization peak: bis-aminoquinoline urea, Put: putrescine, Stn: serotonin, Cad: cadaverine, Tra: tyramine, Tpa + Pha: tryptamine and 2-phenylethelamine.
